# Risk factors for metastasis and survival of patients with T1 gastric neuroendocrine carcinoma treated with endoscopic therapy versus surgical resection

**DOI:** 10.1007/s00464-022-09190-1

**Published:** 2022-05-03

**Authors:** Hua Ye, Yuan Yuan, Ping Chen, Qi Zheng

**Affiliations:** 1 Department of Gastrointestinal and Hernia Ward, HwaMei Hospital, University of Chinese Academy of Sciences, No. 41 Xibei Street, Ningbo, Zhejiang China; 2 Ningbo Institute of Life and Health Industry, University of Chinese Academy of Sciences, Ningbo, China; 3 Key Laboratory of Diagnosis and Treatment of Digestive System Tumors of Zhejiang Province, Ningbo, China

**Keywords:** Endoscopic treatment, Surgery, Metastasis, Survival, Gastric neuroendocrine carcinoma

## Abstract

**Background:**

Gastrectomy with lymphadenectomy is recommended for early gastric Neuroendocrine carcinoma (G-NEC). We attempted to determine the prevalence and risk factors of metastasis of T1 G-NEC and compare the long-term survival of patients after receiving endoscopic therapy (ET) and radical surgery.

**Methods:**

In this study, 205 patients in total with T1 G-NEC were collected from the Surveillance Epidemiology and End Result database. Independent predictors of metastasis were identified by Logistic regression analysis, followed by the calculation of both cancer-specific survival (CSS) and overall survival (OS).

**Results:**

Twenty-five patients (12.2%) were burdened with metastasis at initial diagnosis, with a prevalence of 5.2% (3/58) in mucosa lesions and 16.2% (16/99) in submucosa lesions (*P* = 0.045). No metastasis was detected in lesions with mucosa involvement and tumors ≦ 20 mm (0%, 0/49). The most significant risk factor for metastasis was tumors > 20 mm [odds ratio (OR) 18.64; 95% confidence interval (CI) 4.01–86.68; *P* < 0.001]. For patients with T1N0M0 G-NEC who received ET or surgery, the 10-year OS was similar between the mucosa extension and submucosa extension groups, which was 91.56% in ET group vs 87.50% in surgery group (*P* = 0.62) and 57.33% vs 77.83% (*P* = 0.11), respectively. In addition, the 10-year CSS was also similar between the mucosa extension and submucosa extension groups with 97.30% in ET vs 100% in surgery (*P* = 0.51) and 97.62% vs 86.49% (*P* = 0.65).

**Conclusions:**

In G-NEC, tumors > 20 mm were considered as the most significant risk factor for metastasis. ET seemed adequate for the lesions with mucosa involvement and size ≦ 2 cm.

Neuroendocrine neoplasms (NENs) are defined as a group of epithelial neoplasms with predominant neuroendocrine differentiation based on the 2019 World Health Organization (WHO) classification, which can be categorized into two main groups of well-differentiated NETs and poorly differentiated neuroendocrine carcinomas (NECs) [1]. G-NENs account for only 4% of all NENs, with an incidence of 0.3 per 100,000 annually [2]. However, the incidence of G-NENs has remarkably increased over the past decades, due to various of factors, including accumulative diagnosing experience both clinically and pathologically, enhanced physician awareness and endoscopic surveillance [3]. At present, surgical resection (SR) is widely considered as the backbone of potentially curative approach for G-NENs. Therefore, different surgical approaches have been prevalently applied for G-NENs, from traditional open surgery to endoscopic and laparoscopic resection [4]. Especially, endoscopic therapy (ET), instead of surgery, has been increasingly performed to treat G-NETs of early stage. ET for gastric neuroendocrine tumor (G-NETs) is possible when the tumor is less than 20 mm in size with the infiltration no more than muscularis propria [5]. The outcomes of gastric NENs (G-NENs) vary significantly between gastric neuroendocrine carcinomas (G-NEC) and G-NETs. In addition, to our knowledge, studies comparing the therapeutic outcomes between ET and SR in early stage of G-NEC are rare.

Based on Surveillance Epidemiology and End Result (SEER) database, we attempted to determine the prevalence of metastasis in early stage of G-NEC and further reveal risk factors for metastasis. Finally, we also compared the long-term survival between ET and SR.

## Materials and methods

### Study cohort

The SEER database supported by National Cancer Institute (NCI) covers approximately 28% of the US population from different geographic regions (18 cancer registries) from 2004 to 2015. The SEER database is well-known for its collection and recording on comprehensive information concerning tumor incidence and patient survival. After acquiring access to SEER database and gaining institutional approval, we extracted necessary demographic data, tumor characteristics, therapeutic approach and patient survival by utilizing data items and codes based on NAACCR (the last follow-up was December 31, 2018) [6]. Access to SEER database was obtained, and our study gained institutional approval. This study was approved by the institutional ethical review board of HwaMei Hospital, University of Chinese Academy of Sciences, and IRB approval was also obtained.

### Inclusion and exclusion criteria

Eligible patients were included if they: (1) were diagnosed between 2004 and 2015; (2) were 18 years or older; (3) had Neuroendocrine carcinoma (ENC)(8246/3); (4) had active follow-up data; (5) G-NEC was the first or only primary malignancy; (6) had T1 G-NEC (site codes, C16.0-C16.9) and were treated with either ET or SR based on the SEER database. Specifically, ET referred to endoscopic treatment for local tumor excision with pathology specimen. According to the 8th edition of AJCC TNM staging, G-NEC was referred to TNM staging of gastric carcinoma [7]. Tumor with infiltration into lamina propria or submucosa was defined as T1. The exclusion criteria were as follows: (1) patients with survival < 1 month, mostly likely due to peri-operative complications; (2) their pathological samples were unavailable.

### Statistical analysis

The following data were extracted from SEER database, including age at diagnosis, race, gender, year of diagnosis, tumor grade, tumor size, histology, metastasis, survival (months) and death cause. Overall survival (OS) as well as cancer-specific survival (CSS) were used as the endpoints. Fisher’s exact test or Pearson’s test was employed to compare categorical parameters. Additionally, multivariate logistic regression analysis was used to identity risk factors for metastasis of patients with T1 G-NEC, which was displayed as odd ratios (ORs) and 95% confidence intervals (CIs). In addition, multivariate Cox regression analysis was performed to calculate the adjusted hazard ratios (HRs) and 95% CIs. For comparative long-term survival analysis between ET and SR, patients with T1N0M0 G-NEC were collected, who had low metastasis risks and also received either ET or SR. Eligible patients were subsequently categorized into two groups, namely ET and SR. Specifically, we only extracted the most-invasive tumor-directed treatment from the SEER database.

Statistical analysis was performed using SPSS version 23.0 (SPSS Inc., Chicago, IL, USA). Survival curves were plotted by GraphPad Prism 6.0 (GraphPad Software, San Diego, CA). A two-sided *P* < 0.05 was suggestive of statistical significance.

## Results

### Patient features

A total of 205 patients with T1 (confined to mucosa or submucosa) G-NEC were identified (with mean age of 59 ± 14 years; 125 female [61.0%]; 161 white [78.5%]). In addition, 127 (62%), 40 (19.5%) and 38(18.5%) patients had tumors ≦ 10 mm, 10 to 20 mm and > 20 mm in diameters, respectively. One hundred and seventy-two (95.6%) G-NEC showed well or moderate differentiation out of 180 cases with accessible information on tumor grade. Table 1 summarized the specific information on patient demographics and tumor features.

**Table 1:** Association of clinical and pathologic variables of patients with T1 gastric neuroendocrine carcinoma with survival

Characteristic	*n* (%), total *n* = 205	Mean (month) 95% CI	Statistic	*P*
*Age (years)*			6.723	0.01
Up to 59	92	157 (145.35–168.05)		
60+	113	119 (106.58–130.45)		
*Gender*			3.361	0.067
Female	125	149 (138.05–160.41)		
Male	80	117 (103.46–130.44)		
*Race*			0.272	0.873
White	161	142 (131.55–152.80)		
Black	34	129 (111.09–146.93)		
Others^a^	10	108 (74.12–143.65)		
*Year of diagnosis*			3.0	0.083
2004–2009	57	131 (113.35–148.29)		
2010–2015	148	93 (87.92–98.04)		
*Tumor size (cm)*			2.043	0.36
< 1	127	144 (131.92–155.97)		
1–2	40	133 (117.15–149.78)		
2+	38	131 (108.83–154.01)		
Invasion depth			3.83	0.147
Mucosal involvement	58	158 (145.65–170.56)		
Submucosal involvement	99	138 (123.22–152.46)		
Mucosal or submucosal, not otherwise specified	48	113 (96.80–129.74)		
*Grade*			9.092	0.028
Well-differentiated	147	151 (141.35–160.88)		
Moderately differentiated	25	88 (70.99–105.05)		
Poorly/undifferentiated	8	68 (37.96–97.79)		
Unknown	25	96 (75.04–117.20)		
*Metastasis*			6.99	0.008
No metastasis	180	146 (136.45–155.77)		
Metastasis	25	113 (82.77–143.75)		
*Treatment*			0.035	0.852
Endoscopic therapy	117	143 (130.19–155.37)		
Surgery	88	141 (127.12–155.50)		

^a^American Indian/Alaska Native, Asian/Pacific Islander

### Frequency and risk factors for metastasis

Twenty-five patients (12.2%) suffered metastasis at initial diagnosis, with the incidence of 5.2% (3/58) in mucosa lesions and 16.2% (16/99) in submucosa lesions (*P* = 0.045). With the increase of tumor size, the possibility of metastasis was also elevated. To be specific, the metastasis rates were 2.3% (3/127), 17.5% (7/40), and 39.5% (15/38) in tumors with ≦ 10 mm, 10 to 20 mm and > 20 mm diameters, respectively. Metastasis was not detected for lesions with mucosa involvement and tumors in size ≦ 20 mm (0%, 0/49), which rose to 33.3% (3/9) for tumors > 20 mm. Metastasis rate was 5.7% (3/53), 20.7% (6/27) and 41.2% (7/17) for lesions with submucosa involvement and tumor with ≦ 10 mm, 10 to 20 mm and > 20 mm diameters, respectively.

Multivariate logistic regression model showed that the most significant risk factor of metastasis was tumors > 20 mm (OR 18.64; 95% CI 4.01–86.68; *P* < 0.001). The 2010–2015 years of diagnosis (OR 0.167; 95% CI 0.049–0.574; *P* = 0.004, compared to 2004–2009 years of diagnosis), male (OR 3.534; 95% CI 1.046–11.945; *P* = 0.042, compared to female) were also significant risk factors of metastasis (Table 2).

**Table 2:** Factors predicting metastasis of patients with stage T1gastric Neuroendocrine carcinoma in multivariable analysis

Characteristic	Odds ratio (95% confidence interval)	*P* value
*Age (years)*		
Up to 59	Reference	
60+	0.928 (0.306–2.819)	0.896
*Gender*		
Female	Reference	
Male	3.534 (1.046–11.945)	0.042
*Race*		
White	Reference	
Black	1.393(0.374–5.193)	0.622
Others^a^	6.886 (0.946–50.100)	0.057
*Year of diagnosis*		
2004–2009	Reference	
2010–2015	0.167 (0.049–0.574)	0.004
*Tumor size (cm)*		
< 1	Reference	
1–2	5.29 (11.049–26.682)	0.044
2+	18.643 (4.010–86.680)	< 0.001
*Invasion depth*		
Mucosal involvement	Reference	
Submucosal involvement	3.487 (0.609–19.954)	0.160
Mucosal or submucosal, not otherwise specified	2.030 (0.341–12.072)	0.436
*Grade*		
Well-differentiated	Reference	
Moderately differentiated	0.934 (0.207–4.205)	0.929
Poorly/undifferentiated	6.100 (0.625–59.512)	0.120
Unknown	1.806 (0.363–8.978)	0.470

Adjusted for age, gender, race, year of diagnosis, grade, tumor size, and depth of invasion

^a^American Indian/Alaska Native, Asian/Pacific Islander

### Survival analysis based on invasion depth and therapeutic approaches

**Fig. 1:**
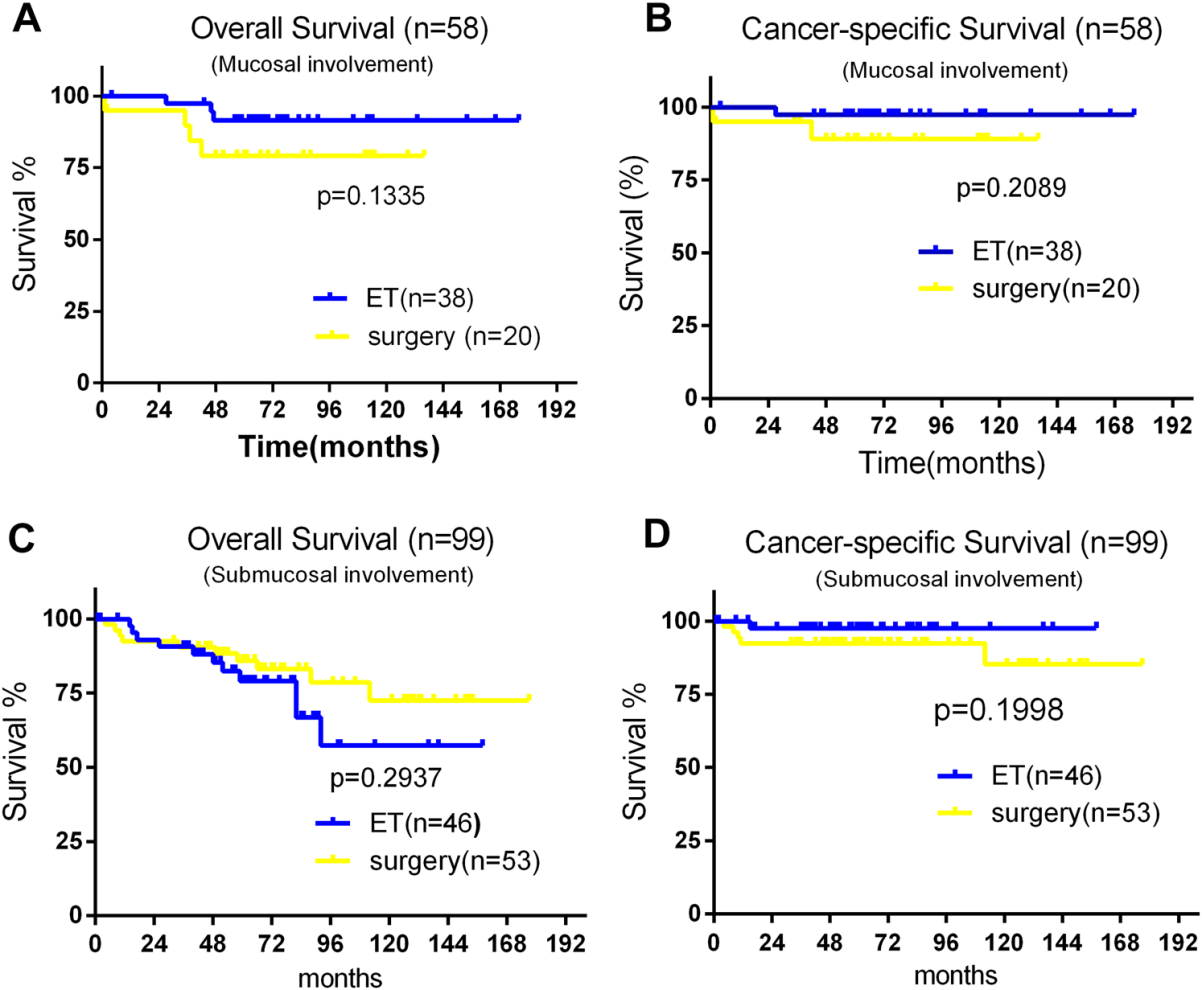
**A** Overall survival (OS) of patients with T1N0M0 gastric-NEC (G-NEC) mucosal involvement was similar between the endoscopic therapy (ET) and surgery groups. **B** cancer-specific survival (CSS) of patients with T1N0M0 G-NEC mucosal involvement was similar between the ET and surgery groups. **C** OS of patients with T1N0M0 G-NEC submucosal involvement between the ET and surgery groups. **D** CSS of patients with T1N0M0 G-NEC submucosal involvement was similar between the ET and surgery groups

For long-term survival analysis, 180 T1N0M0 G-NEC patients at low risks of metastasis receiving either ET or SR were enrolled, including 116 patients (64.4%) undergoing ET and 64 patients (35.6%) receiving SR. Table 3 summarized patient features from two different groups. 31 of 180 patients died during follow-up (71.0 ± 67.0 months). As unadjusted analyses revealed, there was no OS difference between ET and SR groups in mucosa lesion and submucosa lesion subgroups. Figure 1A and C showed that for mucosa extension group, the 10-year OS was 91.56% and 87.50% in ET group and SR group, respectively, which was similar (*P* = 0.62). For submucosa lesions, the 10-year OS was 57.33% and 77.83% in ET group and SR group, respectively (*P* = 0.11).

**Table 3:** Clinicopathologic characteristics of patients with T1N0M0 gastric Neuroendocrine carcinoma in the endoscopic therapy and surgery groups

Characteristic	Endoscopic therapy group (*n* = 116)	Surgery group (*n* = 64)	Statistic	*P*
*Age (years)*			*χ*^2^ = 0.082	0.875
Up to 59	50	29		
60+	66	35		
*Gender*			*χ*^2^ = 1.114	0.33
Female	78	38		
Male	38	26		
*Race*			*χ*^2^ = 6.426	0.04
White	101	46		
Black	12	15		
Others^a^	3	3		
*Year of diagnosis*			*χ*^2^ = 1.275	0.274
2004–2009	24	18		
2010–2015	92	46		
*Tumor size (cm)*			*χ*^2^ = 25.285	< 0.001
< 1	94	30		
1–2	10	23		
2+	12	11		
*Invasion depth*			*χ*^2^ = 5.996	0.051
Mucosal involvement	38	17		
Submucosal involvement	46	37		
Mucosal or submucosal, not otherwise specified	32	10		
*Grade*			*χ*^2^ = 12.545	0.004
Well-differentiated	93	42		
Moderately differentiated	8	13		
Poorly/undifferentiated	1	4		
Unknown	14	5		

^a^American Indian/Alaska Native, Asian/Pacific Islander

It could also be observed from Fig. 1B and D that in the mucosa extension group, 10-year CSS rate was not statistically different between ET group (97.30%) and SR group (100%) (*P* = 0.51). For submucosa lesions category, the 10-year CSS rate was also similar between ET group (97.62%) and SR group (86.49%) (*P* = 0.65).

Adjusted multivariate Cox regression analysis persistently demonstrated that age, year of diagnosis, and tumor grade were significant prognostic factors for both OS and CSS in patients with T1 G-NEC (Table 4).

**Table 4:** Cox regression analysis of OS and CSS in patients with stage T1 gastric neuroendocrine carcinoma

Characteristic	OS		CSS	
	Multivariate analysis		Multivariate analysis	
	HR (95% CI)	*P*	HR (95% CI)	*P*
*Age (years)*				
Up to 59	Reference		Reference	
60+	2.851 (1.330–6.112)	0.007	6.077 (10.260–29.300)	0.025
*Gender*				
Female	Reference		Reference	
Male	1.688 (0.872–3.269)	0.120	4.699 (0.896–24.657)	0.067
*Race*				
White	Reference		Reference	
Black	0.710 (0.277–1.817)	0.475	1.002 (0.193–5.202)	0.998
Others^a^	0.999 (0.222–4.504)	0.999	1.180 (0.079–17.732)	0.905
*Year of diagnosis*				
2004–2009	Reference		Reference	
2010–2015	0.432 (0.208–0.897)	0.024	0.132 (0.027–0.637)	0.012
*Tumor size (cm)*				
< 1	Reference		Reference	
1–2	0.520 (0.198–1.366)	0.185	0.623 (0.087–4.477)	0.638
2+	1.052 (0.441–2.511)	0.910	2.522 (0.480–13.246)	0.274
*Invasion depth*				
Mucosal involvement	Reference		Reference	
Submucosal involvement	1.751 (0.700–4.375)	0.231	0.522 (0.070–3.877)	0.525
Mucosal or submucosal, not otherwise specified	1.988 (0.756–5.225)	0.163	1.163 (0.222–6.093)	0.858
*Grade*				
Well-differentiated	Reference		Reference	
Moderately differentiated	2.391 (0.885–6.460)	0.086	14.028 (1.828–107.652)	0.011
Poorly/undifferentiated	5.0171.25120.113	0.023	78.286 (7.659–800.202)	< 0.001
Unknown	3.0731.2477.570	0.015	9.562 1 (0.510–60.529)	0.016
*Treatment*				
Endoscopic therapy	Reference		Reference	
Surgery	0.844 (0.395–1.805)	0.662	1.375 (0.289–6.533)	0.689

^a^American Indian/Alaska Native, Asian/Pacific Islander

## Discussion

G-NEC, an uncommon disease, accounts for 0.1–0.6% of all types of gastric carcinoma. As a highly aggressive malignancy, G-NEC is characterized by rapid growth, high rate of metastasis and extremely poor prognosis, which is generally more malignant than gastric adenocarcinoma [8]. Due to the recent prevalence of endoscopy for the gastrointestinal tract, there have been an increasing number of asymptomatic early gastric NETs (G-NETs) in clinical practice globally [3]. It is consistent with our findings during all time periods (27.8% in 2004–2009, 72.2% in 2010–2015). It is observed that ET application for T1 lesion has been increasing over time, from 42.1 in 2004–2009, to 62.8% in 2010–2015. Due to the rapidly improved endoscopic techniques, including conventional endoscopic mucosal resection(EMR), endoscopic submucosal dissection (ESD), EMR with ligation device and cap-assisted EMR, higher en bloc and R0 resection rates with lower rates of relevant adverse events are possible, which renders ET as a possible selection for deeper and larger lesions. Moreover, with the accumulative acceptance of ET, the trend of ET is likely to increase [4, 9].

In the 2019 WHO classification, gastrointestinal NETs are classified by the grade of malignancy of each component into NET G1, G2, G3 and NEC [10]. Moreover, there are three types of gastric NETs: type I, NETs generally associated with autoimmune chronic atrophic gastritis; type II, NETs related to Zollinger–Ellison syndrome and multiple endocrine neoplasia type 1 (MEN 1); and type III, aggressive NETs with reportedly sporadic occurrence [11]. National Comprehensive Cancer Network (NCCN) guidelines recommend ET for type I and II gastric NET (≦ 2 cm) [12]. According to European Neuroendocrine Tumor Society (ENETS) Consensus Guidelines and NCCN guidelines for type III gastric NET G3, NEC should be managed with radical surgery, while ET is not recommended even for early-stage lesion. However, ET is an alternative to traditional surgery, which is minimally invasive and increases post-therapeutic quality of life.

As far as we know, it is the first research to compare the long-term survival between ET and SR in T1 G-NEC based on SEER database. Consequently, lesions with mucosa involvement and tumors ≦ 20 mm, metastasis was identified 0%. Multivariate logistic regression analysis revealed that the most significant risk factor of metastasis was tumors > 20 mm (OR 18.64; 95% CI 4.01–86.68; *P* < 0.001). Other risk factors included year of diagnosis (2004–2009 vs 2010–2015) and male. Adjusted multivariate Cox regression analysis persistently revealed that age (≧ 60 years), year of diagnosis (2004–2009 vs 2010–2015) and tumor grade were significant prognostic factors for OS as well as CSS in T1 G-NEC patients. It was found that the proportion of mucosal lesions in year of diagnosis from 2010 to 2015 was higher than that in year of diagnosis from 2004 to 2009 (29.1% vs 26.3%). Therefore, it was likely to cause better prognosis in 2010–2015. Considering the possible recurrence of G-NETs in the long run, abdominal and pelvic multiphasic CT or MRI for at least 10 years after complete resection are recommended by NCCN guidelines [12]. In this study, the long-term (≧ 10 years) OS and CSS rates were similar between ET and SR in T1N0M0 G-NEC patients (Fig. 1A–D), implicating that ET was a promising approach for subjects with low risk of metastasis.

In some researches, ET was recommended for T1 type 3 G-NETs ≦ 10 mm, with G1 grade, With excellent survival despite the possible LNM risk, ET might be an alternative therapeutic option [9, 13]. Our study focused solely on T1 G-NEC, with a large population from the SEER database, and found that the frequency of metastasis of G-NEC mucosa extension and submucosa extension was 5.2% and 16.2%, respectively. Furthermore, the lesions with mucosa involvement and in tumor ≦ 20 mm, metastasis was identified 0%. Toshiaki analyzed that LNM rate of 176 G-NETs confined to SM2 (tumor invasion of or over 0.5 mm into muscularis mucosae) was 14.1% [9]. Although NETs were not classified by tumor grade, their outcomes were similar to ours (LNM rate of 16.2% in submucosa lesions). In our research, metastasis was similarly related to tumor size, tumor invasion and tumor differentiation [14, 15]. NCCN and ENETS guidelines highly recommend EUS to assess tumor infiltration depth, regional lymph node involvement as well as tumor histology before resecting tumor [12, 16]. Thus, in patients with T1 G-NEC and tumor ≦ 20 mm, we should perform EUS before selecting ET.

However, there are certain limitations of this study. To begin with, in this retrospective and observational study, residual confounding is unavoidable. Secondly, inaccessible information on certain variables was recorded by SEER (such as tumor grade, incidental diagnosis, Ki67, the presence of vascular invasion and mitotic rates). Information on other factors such as therapy-associated adverse events, resection margins, endoscopic and surgical procedure details (EMR or endoscopic submucosal dissection; open surgery or minimally invasive techniques), and disease recurrence were inaccessible from SEER database. Otherwise, the lack of records of surgical complications in the SEER database also influenced the complications and overall survival in this study. Additionally, we excluded patients who died within 1 month after surgery to reduce the impact of surgical complications. Chemotherapy treatments are also not available from SEER database. However, we included all patients with early-stage gastric neuroendocrine carcinoma, and chemotherapy that had little effect on overall survival. Anyway, our study enrolls a large population during a long follow-up to reflect real-world clinical outcomes, which is the strength of our study.

In conclusion, in this population-based analysis of T1 G-NEC patients, we demonstrate that tumor > 2 cm is the most significant risk factor of metastasis for T1 G-NEC. ET is likely to be adequate for lesions with mucosa involvement and tumors ≦ 2 cm. Further prospective studies are warranted to evaluate the risk of metastasis and long-term outcomes after different treatment modalities.
